# BASCULE Syndrome in Young Children: Two Cases With Temporally Associated Viral Infections and Diagnostic Challenges

**DOI:** 10.1155/crdm/7922889

**Published:** 2026-10-06

**Authors:** Antonio Prisco, Luigi Coronella, Maddalena Napolitano

**Affiliations:** ^1^ Department of Clinical Medicine and Surgery University of Naples Federico II Naples Italy unina.it

**Keywords:** acrocyanosis, BASCULE syndrome, Bier anaemic spots, orthostatic intolerance, paediatric dermatology, urticaria-like eruption, viral infection

## Abstract

Bier anaemic spots, cyanosis and urticaria‐like eruption (BASCULE) syndrome is a rare vasomotor disorder reported predominantly in adolescents. It is characterized by a distinctive triad of Bier spots, cyanotic discolouration and urticarial lesions, typically triggered by orthostatic stress. We report two cases occurring in young children aged 16 months and 4 years, an age group in which the syndrome is rarely documented and in which the presentation is easily mistaken for vasculitis, drug eruption or sepsis. In both children, the eruption was preceded by a viral infection (Epstein–Barr virus and influenza A, respectively) and both children showed morphological variants that are not classically described: unilateral acral cyanosis with refusal to bear weight in the toddler and a generalized, purpuric‐appearing febrile eruption in the preschool child. In both patients, the cutaneous findings cleared within minutes of recumbency, and neither child had syncope, presyncope or other features of orthostatic intolerance. The eruption resolved with supportive care, and there was no recurrence during follow‐up. The temporal relationship with viral infection observed here is consistent with previous anecdotal reports but does not establish causality, which remains unproven. Recognizing BASCULE syndrome in young children may prevent unnecessary investigations and reduce patient and caregiver anxiety.

## 1. Introduction

BASCULE syndrome is a rare vasomotor disorder characterized by a triad of Bier spots, cyanotic discolouration and urticarial lesions occurring on an erythrocyanotic background [1, 2]. First described in 2016, it is increasingly recognized as a benign yet underdiagnosed condition, particularly in paediatric populations [1, 2]. The pathophysiology remains incompletely understood. Venous pooling induced by orthostatic stress is thought to contribute to erythrocyanosis and Bier spots, while urticarial lesions may result from mast cell degranulation triggered by localized hypoxia [1–3]. Increasing evidence suggests that BASCULE syndrome may fall within the spectrum of autonomic dysfunction, particularly in association with orthostatic intolerance and postural orthostatic tachycardia syndrome (POTS) [2–4]. Several reports have described temporal associations with viral infections, but the available evidence is limited to isolated observations and no causal relationship has been demonstrated [2, 5]. The condition is self‐limiting and generally resolves with conservative measures. However, due to its striking clinical presentation, it may lead to extensive and unnecessary investigations. In the largest review published to date, most of the 21 paediatric cases identified were adolescents between 12 and 19 years of age, with a smaller cluster of cases in neonates and young infants, whereas the toddler and preschool age range remains almost undocumented [2]. Cases in this intermediate age group are of particular interest for three reasons: the child cannot describe pruritus, pain or the postural nature of the eruption, so the diagnostic triad has to be elicited by the examiner; the differential diagnosis at this age is dominated by potentially serious conditions such as vasculitis, meningococcaemia and Raynaud phenomenon, which drives extensive testing, and orthostatic intolerance, which is the association most emphasized in adolescent series, cannot be assessed with the usual tools. We report two cases of BASCULE syndrome in children aged 16 months and 4 years, highlighting clinical variability, the diagnostic pitfalls specific to this age group, and a temporal, not necessarily causal, association with viral infection, including influenza A, which to our knowledge has not previously been reported in this setting.

## 2. Case 1

A 16‐month‐old boy was admitted with episodes of acrocyanosis predominantly affecting the left foot, associated with difficulty walking (Figure 1A,B). His medical history was notable for a recent febrile illness, and he had been receiving amoxicillin/clavulanic acid for 2 days prior to admission. Physical examination revealed urticarial wheals surrounded by pale halos and bluish discolouration of the surrounding skin, which blanched with pressure. Laboratory investigations were unremarkable except for lymphocytosis (7600 lymphocytes; total white blood cell count 12,500/mm^3^). The initial differential diagnosis included postinfectious urticaria, drug‐related eruption and Raynaud phenomenon. The patient was treated with antihistamines and corticosteroids while further investigations were performed, including viral serology, immunological screening, abdominal ultrasound and cardiological evaluation. Dermatological assessment identified a characteristic combination of Bier spots, urticarial lesions and erythrocyanotic changes. Notably, the lesions improved in the recumbent position, supporting the diagnosis of BASCULE syndrome. Epstein–Barr virus serology was positive, suggesting a recent infection. Cardiological evaluation, including electrocardiography and echocardiography, was normal. No syncopal or presyncopal episodes, pallor, sweating, palpitations or feeding‐related cyanotic spells were reported by the parents or observed during admission, and there were no other manifestations suggestive of autonomic dysfunction; formal orthostatic testing was not feasible at this age. Growth and psychomotor development were age‐appropriate and no neurodevelopmental concerns, including attention or hyperactivity problems, had been raised by the parents or by the primary care paediatrician. The patient’s condition resolved spontaneously with rest and reduced physical activity over approximately 2 weeks. At follow‐up visits scheduled at 1, 3 and 12 months, the child remained free of cutaneous lesions and of orthostatic or autonomic symptoms, walking was normal, and no further investigations or treatment were required.

**FIGURE 1:**
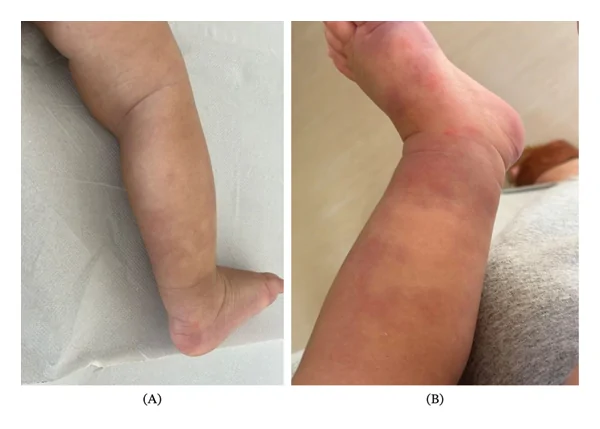
(A, B) Urticarial wheals surrounded by pale halos and bluish discolouration of the surrounding skin in a 16‐month‐old boy.

## 3. Case 2

A 4‐year‐old girl presented with fever and generalized erythematous‐purpuric lesions (Figure 2). Her medical history included recent pharyngitis with tonsillar exudate treated with cefixime, followed by intermittent cough managed with betamethasone. Five days later, she developed erythematous wheals on the buttocks and lower limbs, which progressed despite treatment with cetirizine and additional betamethasone, prompting hospital admission. On admission, she was febrile and exhibited widespread urticarial lesions that were erythematous, warm and centrally darker, resolving with digital pressure. During hospitalization, she developed respiratory symptoms and persistent fever. Laboratory tests showed neutrophilic leucocytosis and elevated inflammatory markers. Dermatological evaluation revealed the characteristic triad of Bier spots, urticarial lesions and erythrocyanotic changes, which resolved upon transition from an upright to a recumbent position, confirming the diagnosis of BASCULE syndrome. Further investigations excluded alternative infectious causes, although a nasal swab tested positive for Influenza A virus. Chest radiography demonstrated interstitial changes consistent with viral infection (the original images were not retrievable in a format suitable for reproduction). Cardiological evaluation, comprising electrocardiography, echocardiography and repeated supine‐to‐standing blood pressure and heart rate measurements, was normal and excluded POTS; the child reported no dizziness and had no syncopal or presyncopal episodes at any time. Neurodevelopmental history was unremarkable and she had never been assessed for, or suspected of having, attention‐deficit/hyperactivity disorder. The patient was treated with antihistamines and oseltamivir. Cutaneous manifestations resolved within 4 days, with progressive improvement in systemic symptoms. She was reassessed at 1, 6 and 12 months after discharge; the eruption did not recur, she remained asymptomatic and no orthostatic intolerance developed.

**FIGURE 2:**
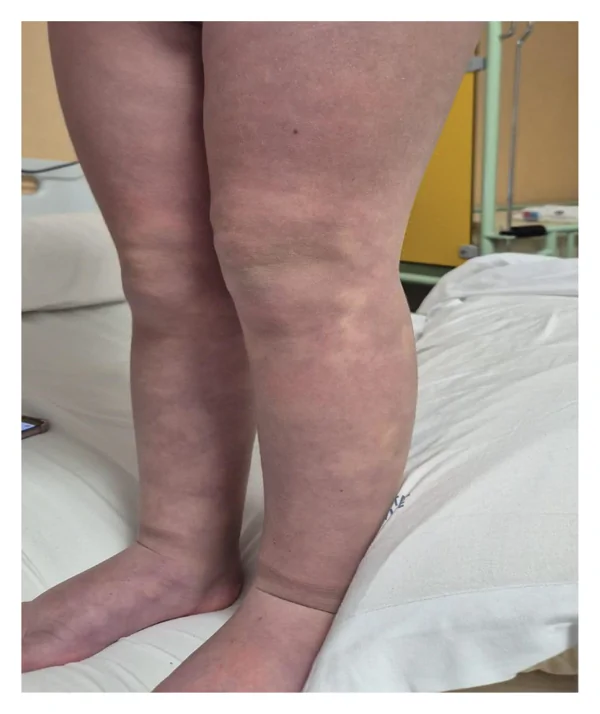
Acrocyanosis and generalized erythematous‐purpuric lesions in a 4‐year‐old girl.

## 4. Discussion

BASCULE syndrome is a benign but under‐recognized paediatric vasomotor disorder characterized by a distinctive clinical triad [1, 2]. The cases presented here illustrate the variability in clinical presentation and the potential for misdiagnosis. The underlying pathophysiology is not fully understood. Venous stasis induced by orthostatic stress is thought to explain the erythrocyanosis and Bier spots, while localized hypoxia may trigger mast cell degranulation, leading to urticarial lesions [1, 3]. The rapid resolution of symptoms in the recumbent position observed in both cases supports this mechanism. The main interest of the present report lies in the age of the two children. In the review by Baurens et al., 21 paediatric cases published between 2016 and 2022 were collated: most patients were adolescents aged 12–19 years and the remaining were newborns or infants in the first months of life [2]. Bessis et al. and Barbé et al. described infants in whom the only manifestation was a transient blue‐white‐red rash of the legs accompanied by unexplained crying after the child had been carried upright for a few minutes [1, 6]; Piroth et al. reported the eruption as an acute painful blue‐white‐red rash of the limbs [7]. Our first patient, aged 16 months, falls between these two described peaks, and his presentation reproduced the paediatric pattern in an incomplete way: the cyanosis was unilateral and confined to one foot, and the equivalent of the pruritus and pain reported by adolescents was a refusal to walk. Our second patient, aged 4 years, is to our knowledge among the youngest reported with a generalized eruption extending to the buttocks and trunk, a distribution previously described mainly in older children and adolescents [8, 9]. Both observations suggest that in preschool children the triad is more often incomplete, asymmetrical or obscured by systemic symptoms and that the postural manoeuvre performed at the bedside, examining the child upright and then recumbent, is the single most useful diagnostic step. The reported morphological and clinical variants of the syndrome are summarized in Table 1.

**TABLE 1:** Reported morphological and clinical variants of BASCULE syndrome.

Component/feature	Classic description	Reported morphological variants and presentations	References
Bier anaemic spots	Multiple 1–5‐mm pale, irregular macules on an erythrocyanotic background of the dependent limb	May form pale halos around the urticarial papules (as in Case 1); occasionally the earliest or predominant sign, with minimal cyanosis	[1–3]
Cyanosis/erythrocyanosis	Diffuse blue‐red discolouration of dependent skin appearing within minutes of standing and clearing in recumbency	Acral and asymmetrical or unilateral involvement in infants and toddlers; “blue‐white‐red” rash of the limbs; may be the presenting sign before urticarial lesions	[1, 6, 7, 10]
Urticaria‐like eruption	Red to orange papules and thin plaques with irregular, oil‐stain‐like borders and a darker centre	Purpuric‐appearing or ecchymotic‐looking lesions mimicking vasculitis; targetoid or erythema‐multiforme‐like lesions; wheals warm to the touch with febrile illness (as in Case 2)	[1, 2, 8]
Distribution	Lower limbs, most often below the knee	Trunk and upper limbs; buttocks and thighs; unilateral single‐limb involvement; generalised eruption	[2, 8, 9]
Trigger	Prolonged standing or sitting with legs dependent	Hot showers and hot environments; being held upright or carried in infants; walking or exertion; no identifiable trigger in a minority of patients	[3, 10]
Associated symptoms	Pruritus, burning pain, paraesthesia and distal oedema	Completely asymptomatic in a minority; in preverbal children, unusual crying, refusal to bear weight or difficulty walking (as in Case 1)	[2, 3, 10]
Autonomic features	Orthostatic intolerance and postural orthostatic tachycardia syndrome (POTS)	Orthostatic hypotension; syncope and presyncope; first‐degree atrioventricular block and QT prolongation; autonomic testing normal in a substantial proportion of patients	[3, 4, 11]
Temporally associated infections	Epstein–Barr virus	SARS‐CoV‐2 (including asymptomatic infection); influenza A (present report); streptococcal pharyngitis preceding the eruption	[2, 5]
Age at presentation	Adolescents aged 12–19 years, female predominance	Bimodal distribution with a second peak in neonates and infants; toddlers and preschool children (present report); adult‐onset cases	[2, 6, 12]
Course and management	Benign and self‐limited; resolves within minutes of recumbency	Recurrent episodes over months to years; variable response to standard‐dose antihistamines; high‐dose bilastine or cetirizine and propranolol reported in selected patients; systemic corticosteroids not indicated	[2, 3, 13]

A second point of interest is the systemic context. Both patients had evidence of a recent viral infection, Epstein–Barr virus in Case 1 and influenza A in Case 2, mirroring earlier observations: Epstein–Barr virus was implicated in the original series [1, 2], and Berrebi et al. described a child in whom BASCULE syndrome followed an asymptomatic SARS‐CoV‐2 infection [5]. Influenza A has not, to our knowledge, been documented previously in association with the syndrome. Nevertheless, respiratory and herpesvirus infections are extremely common in this age group, all published observations are anecdotal, no denominator is available and no case‐control or mechanistic data exist; a shared predisposition, a transient postviral vasomotor instability and simple coincidence are all compatible with the evidence. We therefore regard the association as temporal rather than causal, and prospective collection of infectious data in future series would be needed to test it.

Recent literature has increasingly highlighted an association between BASCULE syndrome and autonomic dysfunction, particularly orthostatic intolerance and POTS [2–4]. In the Mayo Clinic series of 17 paediatric and young adult patients reported by Reinhart et al., autonomic dysfunction was documented in 10 patients, and standing was the trigger in the majority, with hot showers or hot environments in a smaller group [3]. Syncope, presyncope, orthostatic hypotension and conduction abnormalities such as first‐degree atrioventricular block have all been reported in adolescents with the syndrome [4, 11]. Although neither of our patients demonstrated features of orthostatic intolerance, no syncope or presyncope, normal cardiological evaluation in both and exclusion of POTS in Case 2, this association underscores the importance of considering cardiovascular evaluation in selected cases, particularly in adolescents and whenever the history suggests dizziness, fatigue, palpitations or transient loss of consciousness. It should be noted that the absence of autonomic features in our patients may partly reflect their age: standardized active standing and head‐up tilt testing are not validated in toddlers, so a mild degree of orthostatic intolerance cannot be formally excluded. Similarly, neither child was evaluated for attention‐deficit/hyperactivity disorder. Neurodevelopmental and psychiatric comorbidity, including attention deficits, have been described in adolescents and adults with POTS and other dysautonomias, but no such association has been reported for BASCULE syndrome, and a formal ADHD assessment is neither applicable at 16 months nor indicated at 4 years in the absence of parental or paediatrician concern, both of which were absent here. Systematic neurodevelopmental screening in older paediatric cohorts would nonetheless be a reasonable direction for future studies. Diagnosis is primarily clinical and relies on recognition of the characteristic triad and its orthostatic nature. Awareness of this condition is essential to avoid unnecessary diagnostic procedures and inappropriate treatments; in both of our patients, the diagnosis was reached only after an extensive and ultimately unrevealing work‐up, a pattern repeatedly described in the literature and one of the main arguments for wider recognition of the syndrome [2]. Management is generally conservative. Antihistamines may provide symptomatic relief, although their efficacy is variable, and systemic corticosteroids are not routinely indicated; high‐dose bilastine or cetirizine and, in patients with documented autonomic involvement, propranolol have been reported to be of benefit in selected adolescents [3, 13]. Finally, the psychosocial impact on patients and families should not be underestimated. Prompt diagnosis can significantly reduce anxiety and prevent extensive investigations. The increasing number of reported paediatric cases likely reflects improved recognition rather than a true increase in incidence. In summary, these cases extend the reported age range of BASCULE syndrome into the toddler and preschool years, document two morphological variants, unilateral acral cyanosis presenting as refusal to walk and a generalized febrile purpuric‐appearing eruption, and describe influenza A as a newly observed temporally associated infection. Early recognition, based on the simple bedside observation that the eruption clears with recumbency, is essential to avoid unnecessary investigations and to reassure families. The association with preceding viral infection is temporal and, on current evidence, of undetermined causality; larger prospective series with systematic virological and autonomic assessment are needed to clarify it. Uncontrolled follow‐up of two patients over 12 months also limits any conclusion about long‐term prognosis, although the sustained remission observed here is consistent with the benign course reported elsewhere.

## Author Contributions

Methodology, supervision: M.N.; writing–original draft and data collection: L.C. and A.P.

## Funding

The authors have nothing to report. Open access publishing facilitated by Universita degli Studi di Napoli Federico II, as part of the Wiley ‐ CRUI‐CARE agreement.

## Disclosure

All authors read and approved the final version of the manuscript.

## Ethics Statement

Ethical approval was not required, as the study involved retrospective data collection without deviation from routine clinical care.

## Consent

The parents of the patients, being minors, have provided written consent for the publication of the images and for the use of their anonymized clinical data during standard clinical visits.

## Conflicts of Interest

The authors declare no conflicts of interest.

## Data Availability

Data that support the findings of this study are available from the corresponding author, upon reasonable request.

## References

[1] Bessis D. , Jeziorski É. , Rigau V. , Pralong P. , and Pallure V. , Bier Anaemic Spots, Cyanosis With Urticaria-Like Eruption (BASCULE) Syndrome: A New Entity?, British Journal of Dermatology. (2016) 175, no. 1, 218–220, 10.1111/bjd.14589. 27016170

[2] Baurens N. , Briand C. , Giovannini-Chami L. et al., Case Report, Practices Survey and Literature Review of an Under-Recognized Pediatric Vascular Disorder: the BASCULE Syndrome, Frontiers in Pediatrics. (2022) 10, 10.3389/fped.2022.849914. PMC9021422 35463901

[3] Reinhart J. P. , Bangalore Kumar A. , Casanegra A. I. et al., Bridging the Gap in BASCULE Syndrome: A Retrospective Case Series of a Recently Described Clinical Entity, Pediatric Dermatology. (2024) 41, no. 1, 46–50, 10.1111/pde.15470. 38014569

[4] Guillen-Climent S. , Porcar S. S. , Pons Benavent M. , García-Vázquez A. , and Martín J. M. , BASCULE Syndrome Associated With Presyncopal Episodes and First-Degree Auriculoventricular Block, International Journal of Dermatology. (2021) 60, no. 3, 400–402, 10.1111/ijd.15568. 33797752

[5] Berrebi D. , Farmer W. , and Zinn Z. , BASCULE Syndrome in a Child with Prior Asymptomatic COVID-19 Infection, Pediatric Dermatology. (2021) 38, no. 5, 1342–1344, 10.1111/pde.14821. 34595767

[6] Barbé J. , Escobar G. , and Bursztejn A. C. , Bier Anemic Spots, Cyanosis with Urticaria-Like Eruption (BASCULE) Syndrome: A Simple Benign Pathology, but Probably Underdiagnosed, International Journal of Dermatology. (2021) 60, no. 9, 1163–1164, 10.1111/ijd.15452. 33611787

[7] Piroth M. , Guenot L. M. , and Barbarot S. , Acute Painful Blue-White-Red Rash of the Limbs: BASCULE Syndrome, International Journal of Dermatology. (2020) 59, no. 6, 749–750, 10.1111/ijd.14799. 32043279

[8] Jiménez-Gallo D. , Collantes-Rodríguez C. , Ossorio-García L. , Báez-Perea J. M. , and Linares-Barrios M. , Bier Anaemic Spots, Cyanosis with Urticaria-Like Eruption (BASCULE) Syndrome on Trunk and Upper Limbs, Pediatric Dermatology. (2018) 35, no. 5, e313–e315, 10.1111/pde.13566. 29943837

[9] Saratani A. , Seo T. , Watanabe M. , Katayama S. , Ito T. , and Ujiie H. , Bier Anemic Spots, Cyanosis, and Urticaria-like Eruption (BASCULE) Syndrome in a 13-Year-Old Boy With Confirmed Venous Reflux, The Journal of Dermatology. (2026) 53, no. 6, 10.1111/1346-8138.70257. 41951550

[10] Bortoluzzi T. and Silver S. , BASCULE Syndrome: A Report of Three New Cases with Various Associations, SAGE Open Medical Case Reports. (2023) 11, 10.1177/2050313X231152062. PMC9926387 36798678

[11] Danescu S. , Baican C. , Chiorean R. , Filip M. , Cismaru G. , and Baican A. , Orthostatic Hypotension Revealed by BASCULE Syndrome, European Journal of Dermatology. (2018) 28, no. 3, 393–395, 10.1684/ejd.2018.3260. 29619996

[12] Stewart J. M. , Boris J. R. , Chelimsky G. et al., Pediatric Disorders of Orthostatic Intolerance, Pediatrics. (2018) 141, no. 1, 10.1542/peds.2017-1673. PMC5744271 29222399

[13] Cunningham L. , Dvorakova V. , Browne F. , and Irvine A. D. , High-Dose Bilastine for the Treatment of BASCULE Syndrome, Clinical and Experimental Dermatology. (2021) 46, no. 2, 357–358, 10.1111/ced.14377. 32658312

